# A Multidimensional Health Indicator Based on Autoregressive Power Spectral Density for Machine Condition Monitoring

**DOI:** 10.3390/s24154782

**Published:** 2024-07-23

**Authors:** Roberto Diversi, Nicolò Speciale

**Affiliations:** Department of Electrical, Electronic and Information Engineering, University of Bologna, Viale del Risorgimento 2, 40136 Bologna, Italy; nicolo.speciale@unibo.it

**Keywords:** condition monitoring, fault diagnosis, data-driven methods, autoregressive modeling, multidimensional health indicator, Fourier synchrosqueezing transform, spectral distances, signal processing

## Abstract

Condition monitoring (CM) is the basis of prognostics and health management (PHM), which is gaining more and more importance in the industrial world. CM, which refers to the tracking of industrial equipment’s state of health during operations, plays, in fact, a significant role in the reliability, safety, and efficiency of industrial operations. This paper proposes a data-driven CM approach based on the autoregressive (AR) modeling of the acquired sensor data and their analysis within frequency subbands. The number and size of the bands are determined with negligible human intervention, analyzing only the time–frequency representation of the signal of interest under normal system operating conditions. In particular, the approach exploits the synchrosqueezing transform to improve the signal energy distribution in the time–frequency plane, defining a multidimensional health indicator built on the basis of the AR power spectral density and the symmetric Itakura–Saito spectral distance. The described health indicator proved capable of detecting changes in the signal spectrum due to the occurrence of faults. After the initial definition of the bands and the calculation of the characteristics of the nominal AR spectrum, the procedure requires no further intervention and can be used for online condition monitoring and fault diagnosis. Since it is based on the comparison of spectra under different operating conditions, its applicability depends neither on the nature of the acquired signal nor on a specific system to be monitored. As an example, the effectiveness of the proposed method was favorably tested using real data available in the Case Western Reserve University (CWRU) Bearing Data Center, a widely known and used benchmark.

## 1. Introduction

The Prognostics and Health Management (PHM) of machines is gaining more and more importance in the industrial world, especially for firms adopting the main concepts of Industry 4.0 like smart factory and intelligent manufacturing [1,2,3,4]. In this context, condition monitoring (CM) is a crucial aspect, as it plays a significant role in the reliability, safety, and efficiency of industrial operations [5,6]. Condition monitoring, which refers to the tracking of industrial equipment’s state of health during operations, is in fact the basis of modern maintenance strategies like condition-based maintenance (CBM) and predictive maintenance (PM) [1]. The former is usually triggered when a monitored device reaches a certain level of degradation, while the latter relies on the component’s predicted level of deterioration in time. CBM and PM have overcome the drawbacks of corrective and preventive maintenance and allow maintaining a competitive edge and ensuring operational continuity. In fact, these maintenance strategies schedule maintenance activities based on the actual condition of the machinery, as determined by CM data processing.

The information on the health status of machinery, upon which CBM and PM are built, is continuously or periodically assessed by CM through the measurement and analysis of various operational parameters like vibration, temperature, current, and pressure. This information allows detecting equipment changes due to wear, deterioration of components, or other anomalies that, in turn, may indicate the beginning of a fault occurrence. After the data acquisition step, involving the definition of the machine’s critical components and the choice of the sensors to be installed, the central step of CM is data processing [4,5,7]. Data processing consists in extracting, selecting, and (possibly) reducing features from the raw data acquired by sensors. The selected features are then exploited to build health indicators that are able to reveal the health status of the machine’s components. The health indicator is the basis of the machinery fault diagnosis and plays a fundamental role in the last phase of CBM and PM, which is maintenance decision making [1].

The methods employed in the data processing CM step can be divided into three main categories, depending on how much they exploit the physical knowledge related to the monitored system: model-based, data-driven, and hybrid [1,2,8]. Model-based methods rely on physical modeling to build mathematical approximations of increasing degrees of complexity to characterize systems’ input/output behavior. On the contrary, data-driven methods exploit signals measured onboard the system, mainly by means of signal processing and machine learning (or deep learning) techniques. They extract patterns from the available measured data to characterize the status of the machinery. Hybrid methods combine model-based and data-driven approaches. The data-driven approach has become the most used practice in the industry, thanks also to the ever-increasing availability of raw sensor data [5,9].

Many data-driven methods are based on statistical signal processing techniques [4,7,8]. Among them, AutoRegressive (AR) modeling of the measured signals (raw or preprocessed) is a very interesting approach. AR models are one of the most popular tools for time series analysis and spectral estimation [10,11], and they also prove to be a very effective tool for condition monitoring and fault diagnosis [12,13,14,15,16,17,18,19,20]. The widespread use of this class of models is mainly due to the existence of simple and robust algorithms for their identification, the easy implementation of online estimation algorithms, and the high accuracy of the associated spectral estimates. Another feature that makes AR modeling particularly suitable for condition monitoring is its versatility with regard to the type of acquired signal (e.g., vibration, current, torque, temperature) and to the type of machine component to be monitored (e.g., roller bearings, gearboxes, electrical motors, shafts). It is also worth stressing that AR-based techniques are not computationally demanding, so they can be successfully adopted in edge-computing condition monitoring [19,21].

An effective way to perform the CM task with AR models consists in exploiting the associated AR power spectral density (PSD) because of its high sensitivity to signal changes [14,19,20]. Moreover, the use of the AR spectrum makes the diagnostic process easier with regard to the use of the Fourier spectrum [12,16,17,22]. The AR PSD-based approach relies on two main steps: (i) identification of an AR model and the associated AR PSD based on data collected under normal (healthy) operating conditions, and this AR PSD will be considered as the reference PSD; (ii) subsequently, the AR model is continuously or periodically updated using new measured data and the associated PSD is compared with the reference one in order to detect changes in the machine’s behavior. In this paper, we propose a multidimensional health indicator based on AR PSD and the symmetric Itakura–Saito spectral distance, which is used to compare the current PSD with the reference one. It is worthwhile noting that spectral distances are more sensitive to signal changes and more theoretically sound w.r.t. other distances like the Euclidean distance between the AR model coefficients [23].

Despite the very good properties of spectral distances, two limitations may arise. First, the distance is computed by considering the whole spectra; this could make it more difficult to detect incipient or subtle faults as the difference between faulty and normal spectra could manifest only in specific frequency bands; in these cases, the use of the whole frequency content would hide the presence of the fault. Second, the spectral distance is a scalar variable; therefore, it is suitable for fault detection and anomaly detection but not for fault isolation unless the number of faults to be classified is very low. Based on these considerations, we propose to extract a multidimensional indicator from the estimated AR spectrum. To do this, the estimated AR PSDs are divided into a proper number of bands, and the comparison between the current PSD and the reference one is performed band by band, thus obtaining a multidimensional health indicator, called multidimensional symmetric Itakura–Saito spectral distance (MSISSD). Starting from the time–frequency description of the data collected under healthy operating conditions, the definition of the frequency bands is performed by exploiting the properties of the Fourier Synchrosqueezed Transform (FSST).

The effectiveness of the proposed health indicator in condition monitoring has been tested on real data available in the Case Western Reserve University (CWRU) Bearing Data Center [24], which is often used to test new techniques in bearing fault diagnosis [25,26]. This choice is for illustrative purposes only, since the described procedure is related neither to a specific measured signal nor to a specific machine’s component. The obtained results are very promising, as the MSISSD indicator is able to overcome the above-mentioned limitations associated with scalar spectral distances.

The rest of this paper is organized as follows. Section 2 briefly recalls AR models and their identification and describes the derivation of the AR PSD-based multidimensional health indicator. Section 3 describes the proposed CM procedure, including the frequency bands definition through FSST, the AR order selection, and the online monitoring procedure. The results obtained by applying the method to the CWRU dataset are discussed in Section 4. Section 5 concludes the paper with some final comments.

## 2. A Multidimensional Health Indicator Based on AR Spectrum

As mentioned in Section 1, autoregressive models are particularly suitable to represent signals collected from sensors in order to detect changes in the system’s behavior. The measured signal y(t) will thus be described by means of a *p*-order autoregressive (AR) process:

(1) $$y\left(t\right)+a_{1}\;y\left(t-1\right)+\cdots +a_{p}\;y\left(t-p\right)=w\left(t\right),$$

where w(t) is a zero mean driving white noise with variance $\sigma_{w}^{2}$. By introducing the polynomial

(2) $$A\left(z^{-1}\right)=1+a_{1}\;z^{-1}+\cdots +a_{p}\;z^{-p}$$

the AR process can also be seen as the output of an all-pole filter driven by a white noise

(3) $$y\left(t\right)=\frac{w(t)}{A(z^{-1})}.$$

The knowledge of the AR coefficients $a_{1},\ldots ,a_{p}$, the noise variance $\sigma_{w}^{2}$, and the sampling frequency $f_{s}$ allows computing the power spectral density (PSD) S(f) in the frequency domain:

(4) $$S\left(f\right)=\frac{\sigma_{w}^{2}}{|A\left(e^{-j2\pi f/f_{s}}\right){|}^{2}}=\frac{\sigma_{w}^{2}}{|1+\sum_{k=1}^{p}a_{k}\;e^{-j2k\pi f/f_{s}}{|}^{2}}$$

The most popular methods for estimating the AR parameters starting from a set of available measurements ${\left\{y\left(t\right)\right\}}_{t=1}^{N}$ are the least squares (LS) method, the Yule–Walker equations, and the Burg’s method [10,11,27]. Among these approaches, the LS one leads to more accurate estimates, especially when the number of signal samples *N* is not very high [11]. The rationale behind the LS approach, which is also called “covariance method”, consists in finding the AR model associated with the optimal one-step-ahead predictor of the signal y(t). In fact, the LS estimate is found by minimizing the loss function J(θ) given by the sum of the squares of the prediction errors:

(5) $$J\left(\theta \right)=\sum_{t=p+1}^{N}{\left(y\left(t\right)-\varphi^{T}\left(t\right)\theta \right)}^{2}=\sum_{t=p+1}^{N}{\left(y\left(t\right)-\hat{y}\left(t\right)\right)}^{2}$$

where

(6) $$\begin{array}{c} \varphi \left(t\right)={\left[\;-y(t-1)\;\;-y(t-2)\;\cdots \;-y(t-p)\;\right]}^{T} \end{array}$$

(7) $$\begin{array}{c} \theta ={\left[\;a_{1}\;\;a_{2}\;\cdots \;a_{p}\;\right]}^{T} \end{array}$$

and $\hat{y}\left(t\right)=\varphi^{T}\left(t\right)\;\theta$ is the optimal prediction of y(t). The solution is given by

(8) $$\hat{\theta }={\left(\sum_{t=p+1}^{N}\varphi \left(t\right)\varphi^{T}\left(t\right)\right)}^{-1}\sum_{t=p+1}^{N}\varphi \left(t\right)y\left(t\right).$$

An estimate of the additive noise variance $\sigma_{w}^{2}$ can be computed as follows

(9) $${\hat{\sigma }}_{w}^{2}=\frac{1}{N-p}\sum_{t=p+1}^{N}{\left(y\left(t\right)-\varphi^{T}\left(t\right)\hat{\theta }\right)}^{2}=\frac{J(\hat{\theta })}{N-p},$$

where $J(\hat{\theta })$ is computed through (5) by replacing θ with the estimated AR coefficient vector $\hat{\theta }$. As regards the estimation of the AR model order *p*, there are some commonly used approaches, such as final prediction error (FPE), Akaike information criterion (AIC), and minimum description length criterion (MDL) [11,27]. They consist in estimating the AR models of increasing orders and selecting the order *p* corresponding to the minimum of the following cost functions:

(10) $$\begin{array}{cccc} \; & FPE: & \; & p=\min_{k}\left(\frac{N+k}{N-k}J\left({\hat{\theta }}^{k}\right)\right) \end{array}$$

(11) $$\begin{array}{cccc} \; & AIC: & \; & p=\min_{k}\left(N\log J({\hat{\theta }}^{k})+2\;k\right) \end{array}$$

(12) $$\begin{array}{cccc} \; & MDL: & \; & p=\min_{k}\left(N\log J({\hat{\theta }}^{k})+k\;\log N\right) \end{array}$$

where ${\hat{\theta }}^{k}$ is the least squares estimate of an AR model of order *k*.

A very effective way to perform condition monitoring and fault diagnosis by means of AR models consists in exploiting the power spectral density given by (4) [14,19,20,22]. It has been shown that the AR PSD has better diagnostic capabilities with regard to conventional FFT-based techniques [12,16,17,22]. This is mainly due to the fact that, unlike FFT-based methods, the estimated AR spectra do not involve signal windowing, leading to an improved spectral resolution and preventing the introduction of sidelobes effects [11]. To derive a health indicator that is able to check the current status of the system starting from the AR PSD, we adopt the symmetric Itakura–Saito spectral distance SISSD, also known as “cosh” distance [28,29]:

(13) $$SISSD=\frac{1}{N_{f}}\;\sum_{k=1}^{N_{f}}\left(\frac{S_{0}\left(f_{k}\right)}{S(f_{k})}-\log\frac{S_{0}\left(f_{k}\right)}{S(f_{k})}+\frac{S(f_{k})}{S_{0}\left(f_{k}\right)}-\log\frac{S(f_{k})}{S_{0}\left(f_{k}\right)}-2\right)$$

where $N_{f}$ is the number of frequency points, and $S_{0}\left(f\right)$ is a spectrum computed offline during normal operating (healthy) conditions (the “nominal” spectrum), while S(f) is computed online and compared with $S_{0}\left(f\right)$ in order to check if deviations from the healthy state have occurred. Spectral distances are more sensitive to system changes and more theoretically sound with regard to other distances like the root mean square error that compares the Euclidean norms of the parameter vectors $\theta ={\left[\;a_{1}\;a_{2}\;\ldots \;\;a_{p}\;\right]}^{T}$ [23]. It is worthwhile noting that in the Gaussian case, the Itakura–Saito distance corresponds to the Kullback–Leibler divergence up to a multiplicative factor [30], so it also has an interesting statistical interpretation.

Despite the high sensitivity of spectral distances like (13) to anomaly conditions, two limitations may arise: (1) The distance is computed by considering the whole spectra, that is, $f_{k}$ takes values in the range [0,Fs/2], where $f_{s}$ is the sampling frequency. This could make it more difficult to detect incipient or subtle faults as the difference between faulty and normal spectra could manifest only in specific frequency bands. In these cases, the use of the whole frequency content would hide the presence of the fault. (2) The health indicator (13) is a scalar variable; therefore, it is suitable for fault detection and anomaly detection but not for fault isolation, unless the number of faults to be classified is very low. Based on these considerations, we propose to extract a multivariate index from the estimated AR PSD. To do this, the estimated spectra are divided into $N_{b}$ bands, and the comparison between $S_{0}\left(f\right)$ and S(f) is performed band by band, thus obtaining an $N_{b}$-dimensional health indicator as described in the following.

Let $f_{s}$ be the sampling frequency and $S_{0}\left(f\right),0\leq f\leq f_{s}/2$ the nominal AR PSD. According to the procedure described in Section 3.2, $N_{b}$ frequency bands $B_{1},B_{2},\ldots ,B_{N_{b}}$ are selected so that $S_{0}\left(f\right)$ can be divided into $N_{b}$ segments:

(14) $$S_{0}\left(f\right)=S_{0}^{1}\left(f\right)\cup S_{0}^{2}\left(f\right)\cup \cdots \cup S_{0}^{N_{b}}\left(f\right)$$

where

(15) $$\begin{array}{cc} S_{0}^{i}\left(f\right) & =\left\{S_{0}\left(f\right):f_{i}\leq f<f_{i+1}\right\}\;i=1,2,\ldots ,N_{b}-1 \end{array}$$

(16) $$\begin{array}{cc} S_{0}^{N_{b}}\left(f\right) & =\{S_{0}\left(f\right):f_{N_{b}-1}\leq f\leq f_{s}/2\}. \end{array}$$

Similarly, during online health monitoring, the current spectrum S(f) is divided into the same $N_{b}$ bands, and the multivariate symmetric Itakura–Saito spectral distance is computed as follows:

(17) $$MSISSD=\left[\begin{array}{c} ISSD^{1}\\ ISSD^{2}\\ \vdots \\ ISSD^{N_{b}} \end{array}\right]$$

where

(18) $$ISSD^{i}=\frac{1}{N_{f}^{i}}\;\sum_{k=1}^{N_{f}^{i}}\left(\frac{S_{0}^{i}\left(f_{k}\right)}{S^{i}\left(f_{k}\right)}-\log\frac{S_{0}^{i}\left(f_{k}\right)}{S^{i}\left(f_{k}\right)}+\frac{S^{i}\left(f_{k}\right)}{S_{0}^{i}\left(f_{k}\right)}-\log\frac{S^{i}\left(f_{k}\right)}{S_{0}^{i}\left(f_{k}\right)}-2\right)$$

and $N_{f}^{i}$ is the number of frequency points of the *i*-th band, so $\sum_{i=1}^{N_{b}}N_{f}^{i}=N_{f}$. As shown in Section 4, the multivariate index (17) allows one to further increase the sensitivity to signal changes for condition monitoring. Moreover, the MSISSD proves to be a very good feature vector for fault classification.

## 3. Condition Monitoring Procedure

**Figure 1:**
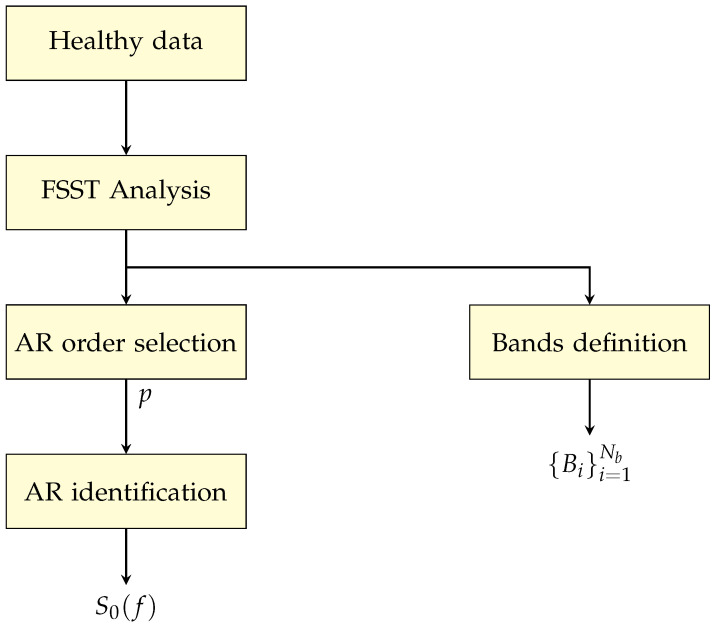
Bands definition and computation of the nominal AR spectrum through healthy data.

**Figure 2:**
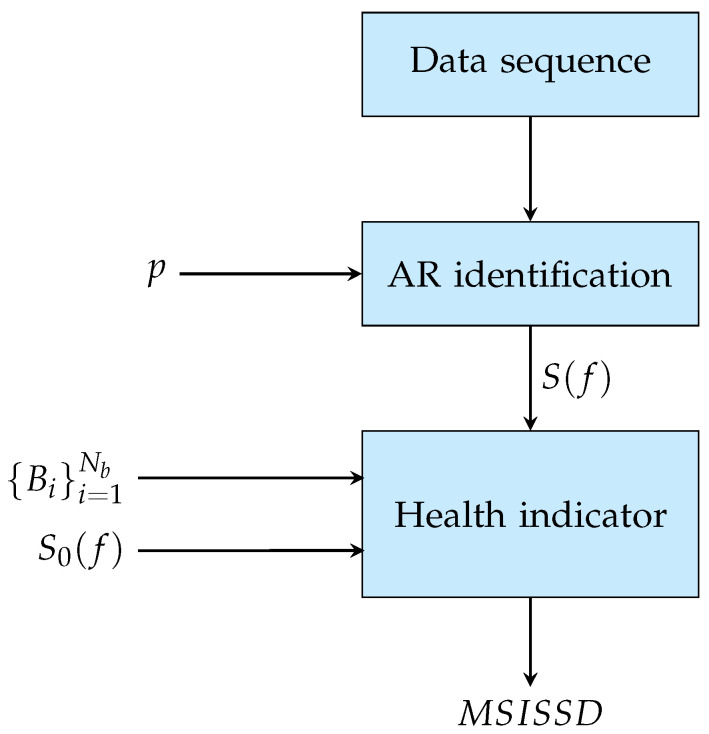
Online monitoring procedure.

The proposed condition monitoring procedure is of general application and can be applied to a very wide set of signals from the monitored system (vibration, current, pressure, torque, …). It can be divided into two distinct phases as depicted in Figure 1 and Figure 2, respectively: (i) bands definition and computation of the nominal AR spectrum and (ii) online monitoring.

The main purpose of the first phase (Figure 1) is to locate the different portions of the spectrum $B_{i}\;\left(i=1,2,\ldots ,N_{b}\right)$ that correspond to the energy distribution in the signal, in the absence of faults (healthy data). Then, the computation of the nominal AR spectrum is performed, and the resulting PSD $S_{0}\left(f\right)$ is used for the online monitoring procedure.

In the second phase (Figure 2), the monitoring procedure can be carried out online. During normal operation, the spectra within each $B_{i}$ band are roughly of the same shape and share the same number of peaks. On the other hand, the appearance of nonidealities due (directly or indirectly) to the presence of a particular defect increases the distance between the nominal and operational spectrum, simplifying the process of identification of the fault occurrence.

### 3.1. Time–Frequency Representation of the Signal

In many real-world operating situations, when using a model-based approach to detect faults in a complex system, there are important parameters that cannot be accurately estimated *a priori*. These include the detection accuracy of frequency components, the extent of damage, and effects due to operating speed, lubrication conditions, or unknown external interference. The proposed approach is based on the recognition of the frequencies that are present in the spectrum during fault-free operation and the bands in which they are located. It is therefore necessary to isolate the most significant instantaneous frequencies in order to define the regions with the highest energy concentration.

**Figure 3:**
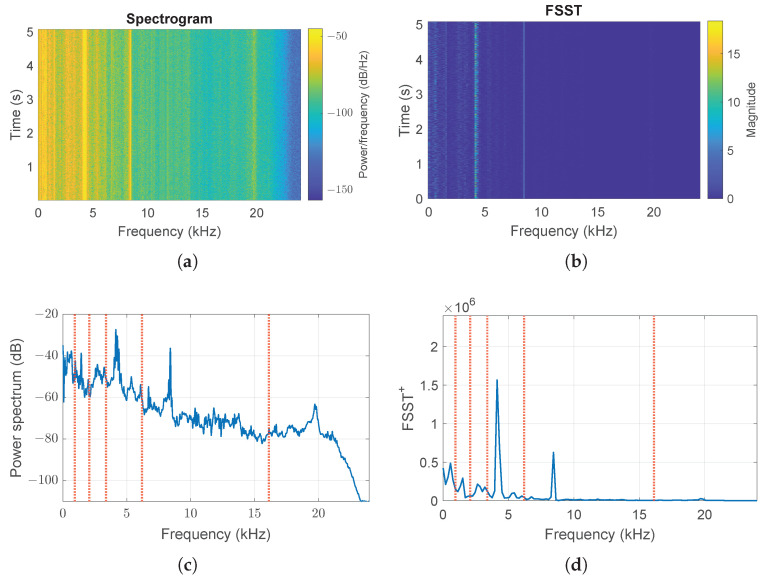
Time–frequency analysis of acceleration signal in the absence of failure and at a maximum speed of 1797 rpm (Healthy0): (**a**) Spectrogram (**b**) magnitude of Fourier Synchrosqueezed Transform, (**c**) Fourier power spectrum, (**d**) integral over time of local instantaneous squeezed frequencies in the TF plane. Vertical red lines identify the boundaries of the bands obtained by the procedure.

The most suitable approach to identify time-varying frequencies is the use of one of the possible time–frequency representations (TFRs) of the signal [31,32]. Among them, the Short-Time Fourier Transform (STFT) [33,34] is widely used for analyzing nonstationary signals in the time–frequency domain. It computes the Fourier Transform of short windowed segments of the real signal y(t) by sliding them along the time axis:

(19) $$STFT\left(t,f\right)=\sum_{\tau =1}^{N_{w}}y\left(\tau \right)g\left(\tau -t\right)e^{-j2\pi f\left(\tau -t\right)/f_{s}}$$

where g(t) is an energy-normalized window sequence of length $N_{w}\leq N$, and *N* is the number of samples. The function of the window is to extract a portion of the finite length of the input signal in such a way that its spectral characteristics are approximately stationary for practical purposes. Spectrogram $S\left(t,f\right)={\left|STFT\left(t,f\right)\right|}^{2}$ is an estimate of the signal PSD at time *t* and frequency *f*. Figure 3a shows the spectrogram of a signal (acceleration) relative to the bearing’s healthy operation at a maximum speed of 1797 rpm (Healthy0: signal 1 in Table 1 related to the motor load of 0 hp). In this TFR, well-defined vertical lines representing stationary frequencies are clearly visible, together with a low-frequency blurred region where weakly nonstationary phenomena occur.

To increase the readability of (19) in the time–frequency plane, the Synchrosqueezing Transformation method [35,36] can be applied to improve its energy concentration by reallocating the values along the frequency axis in the (t,f) plane and constructing a more concentrated time–frequency representation. The procedure is analogous to assigning the total mass of an object to its center of gravity: at each time–frequency point where a spectrogram value is not negligible, the $\left(t,\hat{f}\right)$ coordinates of the local centroid of the TFR are calculated, and the spectrogram value is moved from (t,f) to $\left(t,\hat{f}\right)$, providing a sharper representation. Here, $\hat{f}=\hat{f}\left(t,f\right)$ is the local instantaneous frequency of the signal at time *t*, “filtered” at frequency *f* [36].

The Fourier-based Synchrosqueezing Transform (FSST) [37] is a signal processing technique that provides a concentrated representation of the time–frequency content of a signal by modifying (19) the application of the described technique. It applies a windowed Fourier Transform to segments of the signal and it refines the frequency information by redistributing the energy from neighboring frequencies to the dominant frequency component (*ridge*). By doing so, each column of the FSST(t,f) matrix contains the synchrosqueezed spectrum of the input segment, while the rows correspond to different time instants, and the columns represent frequency bins. The FSST procedure is applied to refine (19), and the magnitude squared is shown in Figure 3b.

It should be noted that, for the proposed approach, the use of the Synchrosqueezing Transform is only aimed at defining the bands $B_{i}$ of interest and not at separating and demodulating the different modes in a multicomponent signal, unlike what is usually carried out. In fact, in the presence of faults, either the magnitude of the spectrum varies or the appearance of particular frequencies in one or more subbands occurs, leading to the identification of a PSD (not necessarily precise, but certainly) different from the nominal one, thus highlighting the presence of a fault.

### 3.2. Partitioning of the Fourier Axis in $N_{b}$ Subbands $B_{i}$

To compensate for (weak) nonstationary effects in the signal and localize the regions of interest, we consider the function

(20) $$FSST^{+}\left(f\right)=\sum_{t}FSST\left(t,f\right)$$

defining a parameter that is somehow related to how much energy is contained within the windowed portions of the signal during the acquisition period. Healthy0 signal function (20) is shown in Figure 3d. By doing so, the peaks located around the dominant “signature frequencies” $N_{b}$$F_{i}^{s}$ become clearly apparent. In this way, bands are defined as the regions of the spectrum that contain a set of fairly pronounced maxima based on their prominence, i.e., how much they differ both in their height and in their position compared with other peaks. An isolated, low peak might be more prominent than a higher one (in absolute value) but not be considered if it belongs to a set of closely spaced higher peaks. We simply define the boundaries $f_{i}$ of each interval as the center between two consecutive maxima: with this set of frequencies, the Fourier spectrum $\left[0,f_{s}/2\right]$ is divided into $N_{b}$ segments, where each band $B_{i}=\left[f_{i},f_{i+1}\right)$ is defined according to

$$f_{1}=0,\;f_{i}=\frac{F_{i}^{s}+F_{i+1}^{s}}{2},\;f_{N_{b}}=f_{s}/2\;i=2,\ldots ,N_{b}-1$$

The number $N_{b}$ is determined by the structure of the signal spectrum, in particular the distribution and the number of instantaneous frequencies found by the synchrosqueezing procedure. Human intervention is very limited: the result depends on the prominence factor and the analysis window used with the STFT (a Kaiser window [38] was chosen for this work, since it maximizes the energy concentration in the main lobe). The choice can be heuristic or, if possible, based on knowledge of physical models, and it is performed only once on the healthy signal. Underestimating the number of bands results in poorly detailed spectrum subdivisions and limits the effectiveness of spectral distance assessment, while excessive subdivisions result in unnecessary computational load without providing better performance. However, tests have shown that the number of bands and their extent depend weakly on the prominence factor and the type of analysis window.

Furthermore, the sum $N_{p}$ of all peaks within the intervals will be used to estimate the order *p* of the AR, a key task for the success of the following identification step. Finally, Figure 3c shows the signal power spectrum and the sub-bands $B_{i}$ considered in the subsequent analysis.

**Figure 4:**
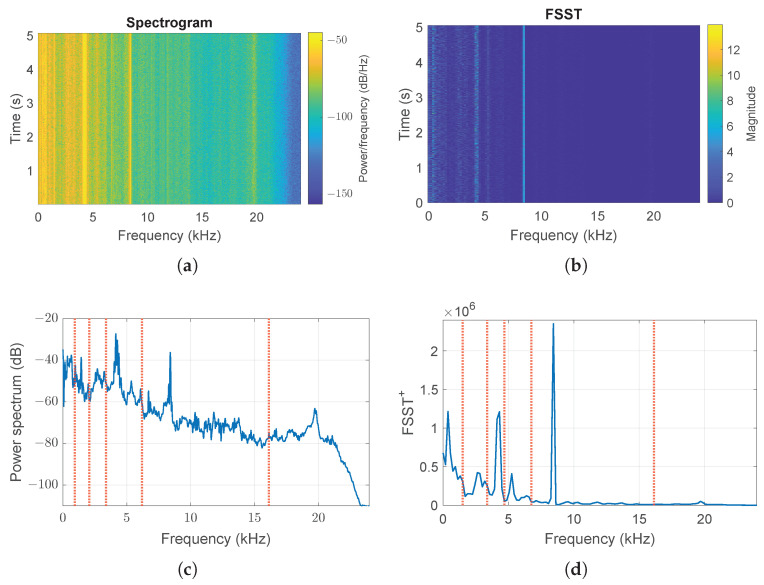
Time–frequency analysis of acceleration signal in the absence of failure and at a speed of 1750 rpm (Healthy2): (**a**) Spectrogram (**b**) magnitude of Fourier Synchrosqueezed Transform, (**c**) Fourier power spectrum, (**d**) integral over time of local instantaneous squeezed frequencies in the TF plane. Vertical red lines identify the boundaries of the bands obtained by the procedure.

The same procedure is also applied to the other available healthy signals for different velocities (1772, 1750, and 1730 rpm), obtaining similar results to those discussed for Healthy0. As an example, signal 1 in Table 1 related to the motor load of 2 hp and velocity 1750 rpm (Healthy2) is shown in Figure 4. Although Figure 3b,d and Figure 4b,d appear very similar, the values of magnitude are different: a different velocity regime produces a similar function (20), and the maxima around the frequencies $F_{3}^{s}=4.21\;\mathrm{kHz}$ and $F_{5}^{s}=8.4\;\mathrm{kHz}$ have different values, but both the total number of bands ($N_{b}=6$) and peaks ($N_{p}=27$) are the same.

### 3.3. Online Monitoring Procedure

As highlighted in Figure 1, the band definition and the estimation of the nominal AR PSD ${\hat{S}}_{0}\left(f\right)$ are performed offline by using a set of data collected under normal (healthy) operating conditions. In particular, the order *p* of the AR model is determined by exploiting both classical criteria like FPE, AIC, and BIC [11,27], as well as the number of peaks $N_{p}$ estimated by the FSST procedure, as detailed in Section 4. The online condition monitoring procedure is then implemented as follows:

1. Collected data of the signal of interest y(t) (vibration, current, etc.) are segmented into (overlapping or not) frames of *N* samples.
2. For each signal frame, an AR model of order *p* is estimated by using the LS approach, and the associated PSD $\hat{S}\left(f\right)$ is computed. The current PSD $\hat{S}\left(f\right)$ and the reference one ${\hat{S}}_{0}\left(f\right)$ are then used to compute the multivariate health indicator MSISSD (17).

Note that this way of exploiting AR modeling follows the same philosophy as the Short-Time Fourier Transform (STFT); for this reason, it has also been called STAR (short-time autoregressive) [39]. In fact, AR identification (and the associated AR spectral estimation) is performed on short segments of measured data and the signal is assumed to be weakly stationary only within each frame. This allows one to quickly detect changes in the frequency content of the signal; therefore, the method can be successfully exploited in applications involving nonstationary signals like speech enhancement [39,40], geophysics [39,41], and predictive maintenance [19].

## 4. Results

**Figure 5:**
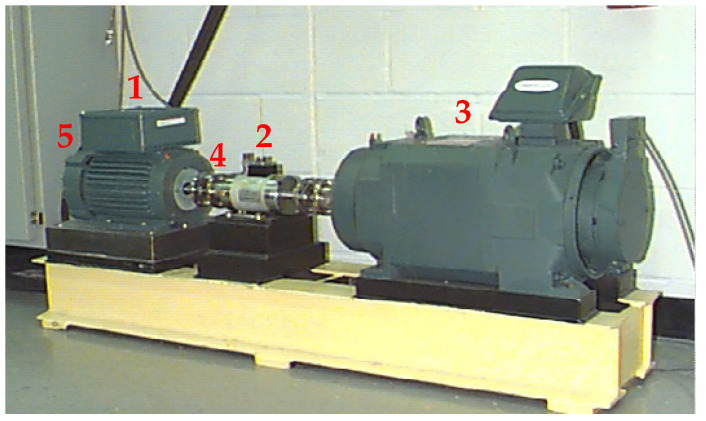
CWRU bearing test rig: (1) electric motor, (2) torque transducer/encoder, (3) dynamometer. Accelerometers are located at the housing of both drive end (4) and fan end (5) bearings.

The effectiveness of the proposed health indicator in condition monitoring has been tested on real data available in The Case Western Reserve University (CWRU) Bearing Data Center [24]. The CWRU dataset is often used as a benchmark for testing new procedures and techniques in bearing fault diagnosis [25,26,42,43,44,45,46]. The test rig shown in Figure 5 consists (see red numbers in the figure) in a 2 hp electric motor (1) driving a shaft, a torque transducer/encoder (2), a dynamometer (3), and control electronics. Single-point faults were artificially introduced to both drive-end (4) and fan-end (5) bearings. The faults are located in the Inner Ring (IRF) and the Ball (BF) rolling element and in three different points on the Outer Ring (ORF). Different fault severities were considered, namely fault diameters of 0.007, 0.014, 0.021, 0.028, and 0.040 inches. The test bearings support the motor shaft and their vibrations are measured by means of piezoelectric accelerometers located at the housing of both drive end (DE) and fan end (FE) bearings. In some tests, accelerometers were also located at the motor-supporting base plate. The test rig operates at a constant speed, and four different motor loads of 0, 1, 2, and 3 hp are considered, corresponding to constant speeds of 1797, 1772, 1750, and 1730 rpm. Vibration data are collected with sampling frequencies of 48 kHz and 12 kHz. It is worth stressing that the detailed analysis of the frequency content of the CWRU-collected signals based on physical considerations highlights the presence of many frequency components not related to the considered faults BF, IRF, and ORF [42]. It was concluded that this is due to mechanical and electromagnetic phenomena. Furthermore, many signals exhibit nonstationary behavior. As a consequence, fault classification methods relying on the true bearing fault frequencies are not able to perform the classification task in some of the collected data sequences [42].

In this paper, the DE vibration data sampled at 48 kHz are considered, including the DE normal baseline (healthy) data, that have also been sampled at 48 kHz [42]. In this case, for every motor load (0,1,2, and 3 hp) 14 classes of collected signals are available [24,42], see Table 1.

A good health indicator should be able to detect as soon as possible deviations from normal behavior and “to follow” the possible increment of the fault severity over time. Therefore, to test the fault detection capabilities of the health indicator (17) in condition monitoring, we apply the procedure described in Section 3 to a set of signals that refer to the normal state and to the different severities of the same fault. In particular, the procedure is applied to the set of signals {1,2,3,4}, {1,5,6,7}, and {1,11,12} (see Table 1) related to the motor load of 0 hp (speed 1797 rpm). Note that each set refers to a different type of fault (BF, IRF, and ORF orthogonal, respectively) and includes the normal (healthy) status and three faulty status corresponding to three different fault sizes (0.007 in, 0.014 in, and 0.021 in) except for ORF orthogonal, where the size of 0.014 inches is not available. For each set, the condition monitoring procedure is implemented as follows:

1. A portion of the healthy signal is used to define the frequency bands, $B_{1},B_{2},\ldots ,B_{N_{b}}$, to select a proper order *p* of the AR model, to estimate an AR model of the selected order, and to compute the associated reference PSD ${\hat{S}}_{0}\left(f\right)$ through (4). Note that this offline step is the same for every set as it involves only the (same) healthy signal.
2. The remaining part of the healthy data and the faulty data sequences are segmented into frames of *N* = 20,000 samples. For each signal frame, an AR model of order *p* is estimated by using the LS approach, and the associated PSD $\hat{S}\left(f\right)$ is computed. The current PSD $\hat{S}\left(f\right)$ and the reference one ${\hat{S}}_{0}\left(f\right)$ are then used to compute the health indicator. Both the scalar spectral distance SISSD (13) (that does not use the frequency bands but only the whole spectrum) and the multidimensional distance MSISSD (17) are considered.

**Figure 6:**
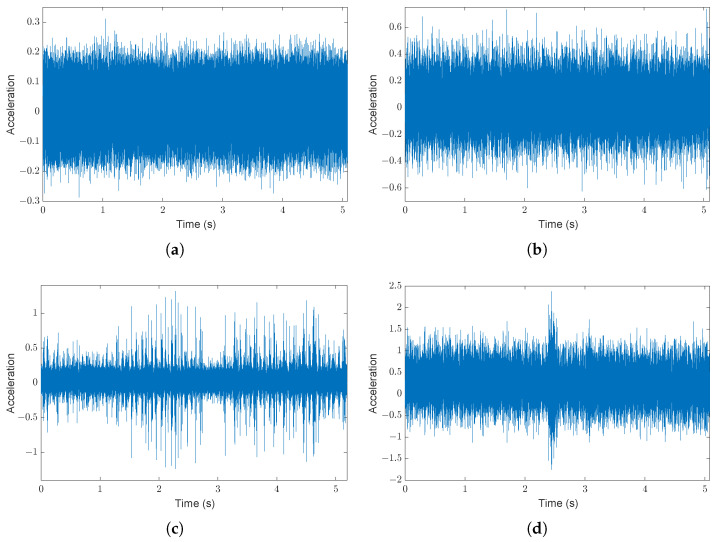
CWRU DE vibration signals sampled at 48 kHz (motor load 0 hp): (**a**) healthy, (**b**) ball fault (0.007 inches), (**c**) ball fault (0.014 inches), (**d**) ball fault (0.021 inches).

**Figure 7:**
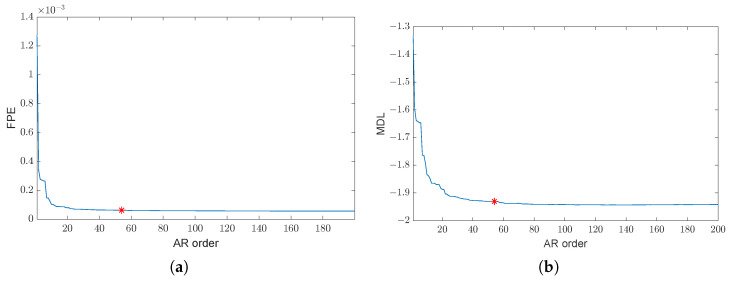
Estimation of the AR order *p*: (**a**) FPE criterion (**b**) MDL criterion. The red star shows the values of FPE and MDL associated with the order p=54, which is double the number of peaks $N_{p}$ estimated through the FSST procedure.

The time evolution of the signals of the first set ({1,2,3,4}), involving healthy and ball fault conditions, is shown in Figure 6. The first phase of the proposed approach (see Figure 1), performed on a portion of the healthy signal (Figure 6a), leads to the definition of the six bands reported in Table 2. The corresponding number of peaks is $N_{p}=27$. By taking into account $N_{p}$ and the selection criteria (10)–(12), we decide to choose the AR order p=54 to double the number of peaks estimated by the FSST (remember that a peak can be associated with a couple of complex conjugate poles in the filter (3)). In fact, on the one hand, the FPE criterion does not exhibit a minimum for orders ranging from 1 to 200, while the MDL criterion leads to a minimum of 135, as can be seen in Figure 7 (the AIC is not reported, as it shows behavior similar to FPE). On the other hand, the criteria tend to stabilize after a strong initial decrease. The order p=54 has therefore been selected for two reasons: (1) the peaks estimated are the ones that “take” the major part of the energy; see Section 3.1; (2) the value p=54, highlighted in red in Figure 7, belongs to the region where the criteria are almost constant (the decrease in MDL from 54 to the minimum 135 is less than 1%). However, other AR models with orders both smaller and larger than 54 were tested. The obtained results are not reported as there is no significant difference compared with those obtained with p=54.

**Figure 8:**
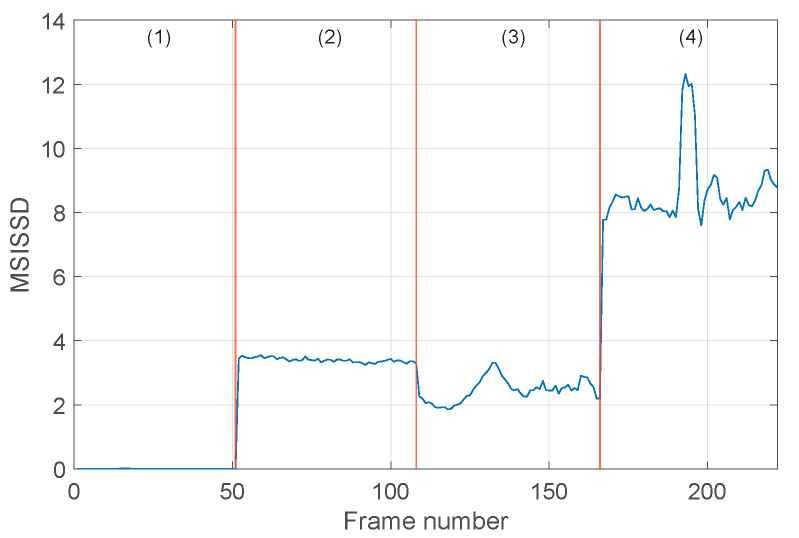
Evolution of the SISSD indicator in the four different conditions associated with the set of signals {1,2,3,4} as a function of the signal frames: (1) healthy, (2) BF (0.007 in), (3) BF (0.014 in), (4) BF (0.021 in).

**Figure 9:**
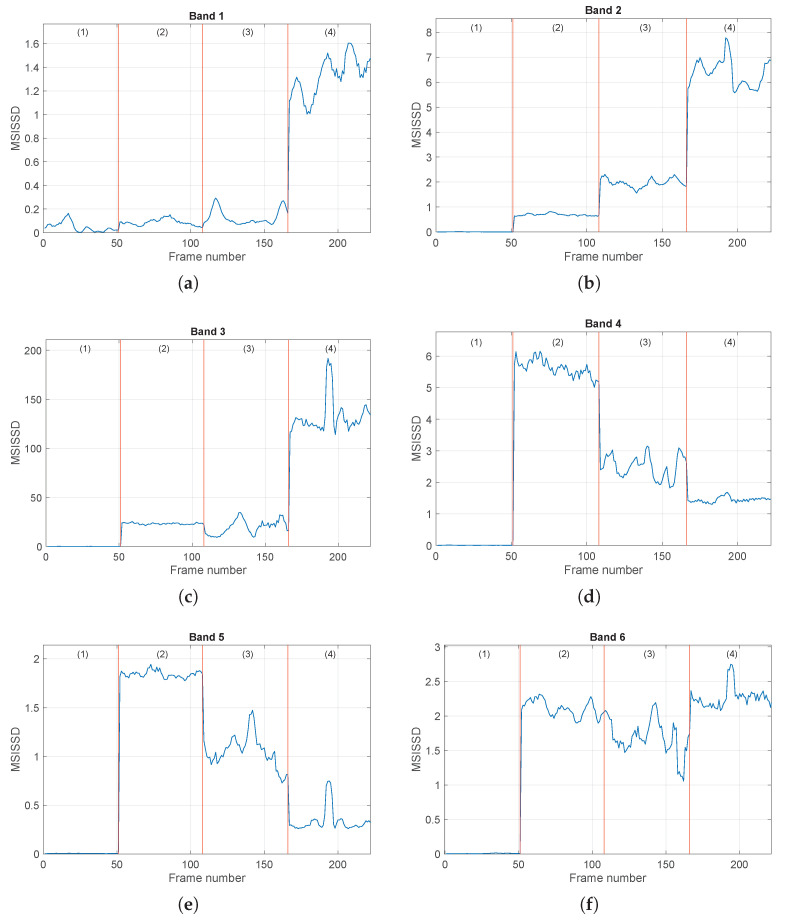
Evolution of the MSISSD indicator in the four different conditions associated with the set of signals {1,2,3,4} for all the defined frequency bands as a function of the signal frames. Subfigures (**a**–**f**) refer to the subbands 1–6 defined in Table 2. The picture associated with Band *i* reports the evolution of the *i*-th entry of the MSISSD indicator. For every picture, the four conditions are (1) healthy, (2) BF (0.007 in), (3) BF (0.014 in), (4) BF (0.021 in).

The online procedure is performed by using the determined bands $B_{i}$ in Table 2, and the selected order and the values of both the indicators SISSD and MISSD are computed. The evolution of the scalar indicator SISSD in the four conditions is reported in Figure 8, as a function of the signal frames. We can see that, during normal conditions, the values of the SISSD are very close to zero, as expected. In fact, in all signal frames, the current PSD $\hat{S}\left(f\right)$ is very close to the reference one ${\hat{S}}_{0}\left(f\right)$, as the healthy signal exhibits a stationary behavior. The ball fault conditions can be clearly distinguished from the healthy ones, and very robust detection thresholds can be defined. It is worth noting the (slightly) nonstationary behavior of the indicator in the third condition and the nonstationary event that occurs in the fourth condition. These behaviors also emerge from the time domain signal waveform; see Figure 6c,d. This figure shows the ability of the spectral distance to detect signal changes because of its high sensitivity. Nevertheless, in this case, the SISSD is not able to discriminate all the different fault severities, in particular the second condition from the third one (0.007 in vs 0.014 in). Figure 9 reports the evolution of the six entries of the multivariate indicator MSISSD, associated with the six estimated bands reported in Table 2. Two important observations can be made: (1) The fault detection ability of the indicator is very good, and the sensitivity is even higher with respect to SISSD, as shown in Figure 9c. (2) The different fault sizes can be clearly distinguished, see Figure 9b,d,e.

**Figure 10:**
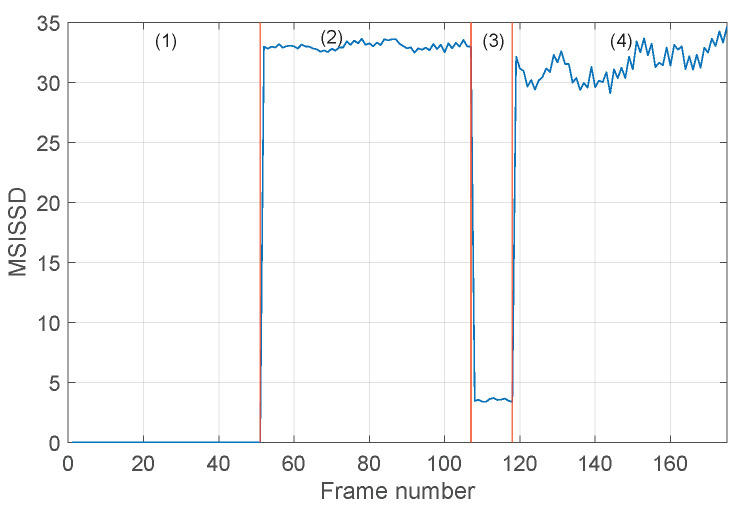
Evolution of the SISSD indicator in the four different conditions associated with the set of signals {1,5,6,7} as a function of the signal frames: (1) healthy, (2) IRF (0.007 in), (3) IRF (0.014 in), (4) IRF (0.021 in).

**Figure 11:**
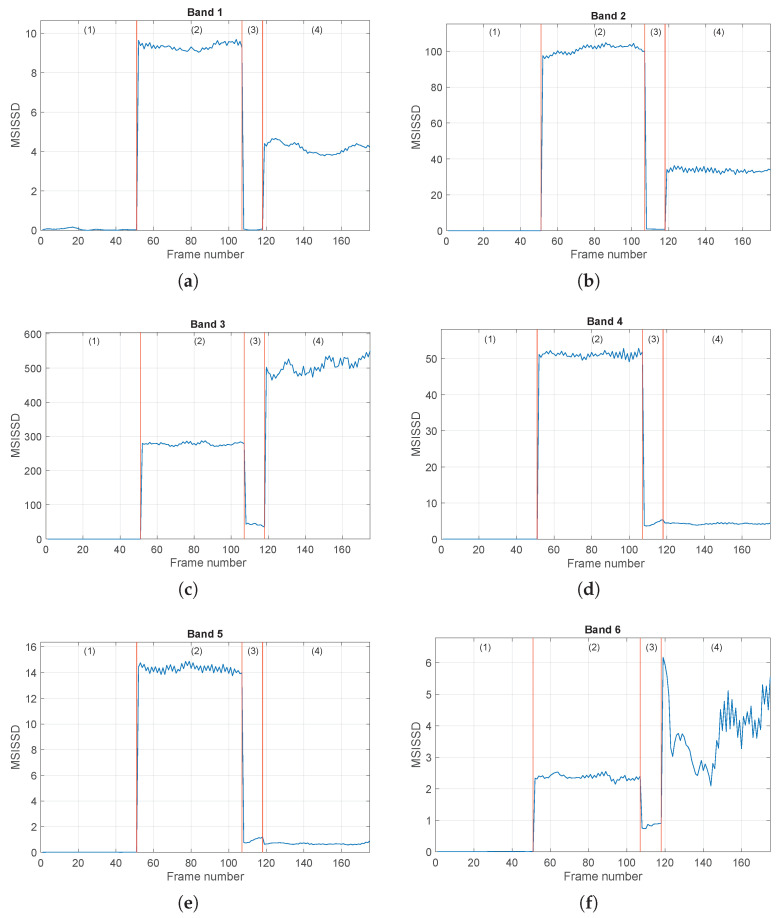
Evolution of the MSISSD indicator in the four different conditions associated with the set of signals {1,5,6,7} for all the defined frequency bands as a function of the signal frames. Subfigures (**a**–**f**) refer to the subbands 1–6 defined in Table 2. The picture associated with Band *i* reports the evolution of the *i*-th entry of the MSISSD indicator. For every picture, the four conditions are (1) healthy, (2) IRF (0.007 in), (3) IRF (0.014 in), (4) IRF (0.021 in).

The procedure is then applied to the set of signals ({1,5,6,7}), concerning healthy and inner ring fault conditions. The obtained results are reported in Figure 10 and Figure 11. Again, the SISSD allows the detection of faulty situations with very high relative distances; see Figure 10. The discrimination of the different fault sizes is, however, not possible, as conditions (2) and (4) present similar levels of the health indicator. On the contrary, these faulty situations are clearly distinguished when the multidimensional indicator is used, see Figure 11. It is worth noting the strange behavior of the third condition (IRF-0.014 in) in all six frequency bands. This is not surprising in light of the analysis performed in [42] on the envelope spectrum of this signal. In fact, it was concluded that the spaces of the pulses of the spectrum, and their modulation seems quite random and not related to inner ring fault and shaft speed.

**Figure 12:**
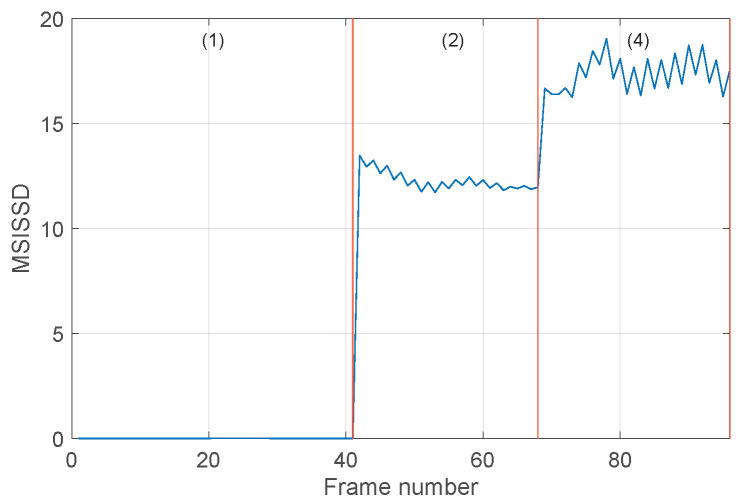
Evolution of the SISSD indicator in the three different conditions associated with the set of signals {1,11,12} as a function of the signal frames: (1) healthy, (2) ORF orthogonal (0.007 in), (4) ORF orthogonal (0.021 in).

**Figure 13:**
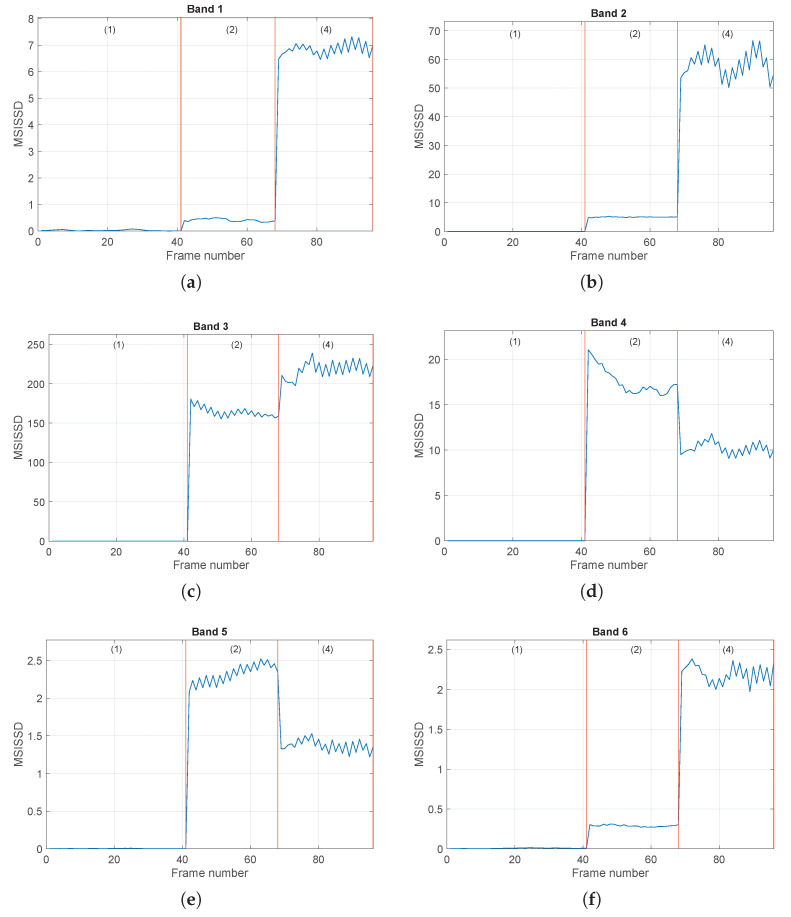
Evolution of the MSISSD indicator in the four different conditions associated with the set of signals {1,11,12} for all the defined frequency bands as a function of the signal frames. Subfigures (**a**–**f**) refer to the subbands 1–6 defined in Table 2. The picture associated with Band *i* reports the evolution of the *i*-th entry of the MSISSD indicator. For every picture, the four conditions are (1) healthy, (2) ORF orthogonal (0.007 in), (4) ORF orthogonal (0.021 in).

The last considered set of signals is {1,11,12}, which includes healthy, ORF orthogonal (0.007 in), and ORF orthogonal (0.021 in). The results are shown in Figure 12 and Figure 13. These figures essentially lead to observations similar to those made for the previous types of faults. Even though in this case the scalar indicator SISSD allows one to distinguish the fault sizes (see Figure 12), the multivariate indicator successfully performs the same task with remarkably higher relative distances, as can be seen by comparing, for instance, Figure 12 with Figure 13a,b.

### Evaluation of the MSISSD Indicator in Fault Classification

In this subsection, we show some preliminary results concerning the performance of the multivariate indicator MSISSD in fault classification. It is worth stressing that the main focus of this paper is not fault classification, and the aim of this subsection is not a complete classification of the CWRU dataset. For this reason, only 48 kHz drive-end data and only one classifier (support vector machine with linear kernel) have been considered.

For each motor load, 0,1,2,3 hp, all the signal classes reported in Table 1 are taken into account, so we have four different multiclass classification problems, each involving 14 classes. For every motor load (speed), the MSISSD-based classification procedure was implemented as follows:

1. A portion of the healthy signal (1 in Table 1) is used to define the frequency bands $B_{1},B_{2},\ldots ,B_{N_{b}}$, to select a proper order *p* of the AR model, to estimate an AR model of the selected order and to compute the associated reference PSD ${\hat{S}}_{0}\left(f\right)$ through (4).
2. The remaining part of the healthy signal and the faulty signals 2,3,…14 are segmented into frames of *N* = 20,000 samples. For each signal frame, an AR model of order *p* is estimated by using the LS approach, and the associated PSD $\hat{S}\left(f\right)$ is computed. The current PSD $\hat{S}\left(f\right)$ and the reference one ${\hat{S}}_{0}\left(f\right)$ are then used to compute the multivariate spectral distance MSISSD (17).
3. At the end of step 2, we have a set of MSISSD points for each of the 14 classes representing the different conditions. For every class, the related MSISSD points are labeled with the corresponding class number and divided into a training set and a validation set. As regards the number of points of the training and validation sets, both the 70%/30% and 50%/50% fractions are considered.
4. The classification task is performed by means of the support vector machine (SVM) classifier with a linear kernel and one-versus-one approach. To this end, the MATLAB [47] function “fitcecoc” was employed.

Steps 3 and 4 are repeated 200 times in order to test 200 different combinations of training and validation points. Therefore, 200×2=400 tests were carried out for every motor load, for a total of 1600 tests. The results are summarized in Table 3, which reports the means of accuracy over 200 runs, the worst accuracy, and the number of runs where 100% accuracy was obtained.

**Figure 14:**
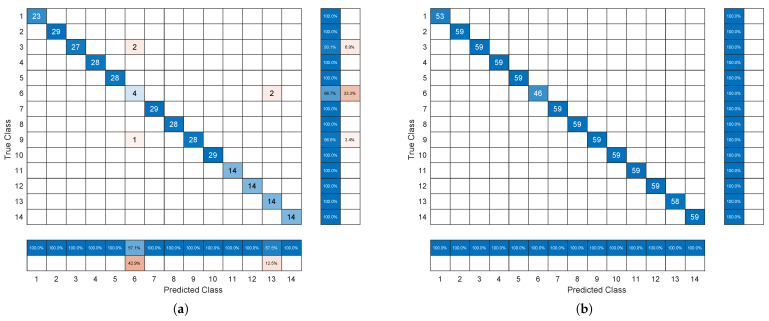
Confusion matrices associated with two classification experiments: (**a**) 0 hp load, 50% training ratio, worst case (accuracy 98.41%), (**b**) 1 hp load, 50% training ratio, accuracy 100%.

Table 3 shows the promising performance of the proposed health indicator, which is also in the fault classification. The MSISSD is able to discriminate both the different types of faults and the different severities of each type of fault. It is also able to classify the different ORF subtypes that are fault centered in the load zone, fault orthogonal to the load zone, and fault opposite to the load zone. It is also worth noting that, if we consider the two macroclasses of healthy (signal 1) and faulty (signals form 2 to 14), both precision and recall are always equal to 1 in all 400 performed tests. This confirms the very good fault detection ability of the MISSD indicator, which is already pointed out in Figure 8, Figure 9, Figure 10, Figure 11, Figure 12 and Figure 13. As already mentioned, the objective of this paper is not CWRU data classification, and the obtained results can be further improved. As an example, Figure 14 compares the confusion matrix of the worst case test (load 0, 50% training ratio) with one of the confusion matrices related to load 1 and 50% training ratio (accuracy always equal to 100%; see Table 3). As it can be seen, the dataset employed for the load 0 hp is unbalanced, in particular, with reference to the 6-th class (IRF 0.014 in), and all the misclassified points are related to this class. As previously mentioned, it was claimed in [42] that this signal exhibits a random behavior not related to a specific type of fault; see also Figure 10 and Figure 11. By repeating the classification experiments taking into account all classes but the 6-th one, the 0-hp row of Table 3 becomes equal to the 1-hp row, i.e., no points are misclassified for both the training ratios. This confirms the negative role played by the sixth signal, even if the overall results are satisfactory.

## 5. Conclusions

A machine condition monitoring procedure based on a new multidimensional health indicator has been described. The proposed indicator relies on the spectrum of the autoregressive model of the signal to be monitored and on the Itakura–Saito spectral distance. The method has the following features:

- The initial parameter setting step is carried out on the healthy signal only once.
- It can be applied to different types of acquired signals (vibration, current, torque, etc.) and to different types of machine components (bearings, gearboxes, shafts, etc.) from real industrial contexts without the need to interrupt the operation of the monitored system.
- It is not designed for a specific case, even though only vibration signals and roller bearings have been considered in this paper. Moreover, the successful use of the AR PSD for other signal/components, like current/bearings [14], vibration/gears [17], PLC torque/shaft [20], can be seen as further case studies where the MSISSD indicator can be successfully applied.
- The multivariable nature of the indicator may improve the detection of subtle faults with respect to spectrum-based scalar indicators.
- It allows one to perform the fault classification task without requiring a huge amount of data, unlike modern data-based approaches.
- There is no need to precisely identify the characteristic frequencies of faults, since it is only important to highlight the emergence of changes in the spectra.
- All steps involve operations that are not computationally critical, so the method can also be adopted as part of monitoring edge-computing conditions, allowing for early detection of failures.

Tests performed using the CWRU dataset show the effectiveness of the approach in detecting signal changes and its suitability for online monitoring and fault diagnosis. The preliminary results obtained in the classification of failures of part of the CWRU dataset are very promising, so the use of the health indicator for fault classification deserves further investigation. Another interesting topic to cover is the application of the method to data collected from industrial contexts.

## Figures and Tables

**Table 1:** Available CWRU vibration data collected at 48 kHz for each motor load (0,1,2,3) [24].

Signal Class Number	Type of Signal
1	Normal (healthy)
2	BF (0.007 in)
3	BF (0.014 in)
4	BF (0.021 in)
5	IRF (0.007 in)
6	IRF (0.014 in)
7	IRF (0.021 in)
8	ORF centred (0.007 in)
9	ORF centred (0.014 in)
10	ORF centred (0.021 in)
11	ORF orhtogonal (0.007 in)
12	ORF orthogonal (0.021 in)
13	ORF opposite (0.007 in)
14	ORF opposite (0.021 in)

**Table 2:** Frequency bands obtained by applying the procedure described in Section 3.2 to a portion of the healthy signal of Figure 6a.

Band Number	Frequency Range (kHz)
1	[00.93)
2	[0.932.06)
3	[2.063.38)
4	[3.386.19)
5	[6.1916.13)
6	[16.1324.00]

**Table 3:** Classification results of the CWRU dataset (48 kHz, DE data). For each motor load, classification problems with 14 classes are solved; see Table 1. For every motor load and training data fraction (70% and 50%), 200 classification experiments were carried out.

Motor Load (hp)	Training Set (%)	Accuracy (Mean)	Accuracy (Worst)	No of Runs with 100% Accuracy
0	70%	99.81%	98.92%	132/200
	50%	99.69%	98.41%	81/200
1	70%	100%	100%	200/200
	50%	100%	100%	200/200
2	70%	99.99%	99.18%	197/200
	50%	99.91%	98.90%	152/200
3	70%	99.84%	99.38%	76/200
	50%	99.82%	99.14%	28/200

## Data Availability

The original data presented in the study are openly available in The Case Western Reserve University Bearing Data Center at https://engineering.case.edu/bearingdatacenter (accessed on 28 June 2024).
